# Using unmanned aerial vehicles (drones) to improve access to blood in low‐ and middle‐income countries: Current challenges and opportunities

**DOI:** 10.1111/vox.70207

**Published:** 2026-02-15

**Authors:** Suvro Sankha Datta, Ritwick Mondal, Shramana Deb, Giuseppe Tortora, Angela Pirri

**Affiliations:** ^1^ Department of Transfusion Medicine Tata Medical Center Kolkata India; ^2^ Department of Neurology Manipal Groups of Hospitals Kolkata West Bengal India; ^3^ Smart Medical Theatre Laboratory, ABzero srl Cascina Pisa Italy; ^4^ Institute of Applied Physics “N. Carrara” IFAC Consiglio Nazionale delle Ricerche Sesto Fiorentino Florence Italy

**Keywords:** accessibility, blood transfusion, challenges, low‐ and middle‐income countries, transportation, unmanned aerial vehicles

## Abstract

Blood transfusion is life‐saving for patients in emergencies, but low‐ and middle‐income countries (LMICs) often face a severe shortage of banked blood. Establishing blood banks in rural areas presents substantial logistical and economic challenges for many LMICs. Furthermore, difficult terrain, inadequate transportation infrastructure and adverse environmental conditions frequently cause delays in delivering essential blood supplies to patients in need. However, the freight transport system is undergoing a significant transformation with the introduction of unmanned aerial vehicles (UAVs), commonly known as drones. This environmentally conscious technology has made remarkable progress in recent times and demonstrates the potential to transport vital medical supplies, including blood and blood components, to remote areas during emergencies. Moreover, the use of artificial intelligence in drones has enhanced their flight safety. In this brief review, we first outline the various types of UAVs available for use, along with their respective configurations and regulatory requirements. Then we point out key problems of transfusion services within resource‐limited regions, which include not having a national blood policy, a mismatch between supply and demand, a lack of robust testing facilities for bloodborne infections and not adhering to the transfusion guidelines. While resolving all challenges may seem unrealistic, we try to explore potential pathways to significantly improve blood accessibility for patients in LMICs through the strategic implementation of UAVs, considering the various barriers and facilitators associated with this innovative approach.

**Highlights**

- Blood transfusion is essential for managing various medical conditions; however, access to blood components remains a substantial challenge in low‐ and middle‐income countries.
- Unmanned aerial vehicles (UAVs) have the potential to increase the capacity and efficiency of healthcare systems by delivering essential blood components to remote areas during medical emergencies and disasters.
- UAV‐based blood delivery can decrease blood product wastage by optimizing distribution and streamlining supply chains.

## INTRODUCTION

Even in the third decade of the 21st century, billions live in parts so remote from the standard clinical facility with a functional blood bank that they can be easily conceived as ‘blood deserts’ [1]. A blood desert is defined as a geographical region where essential clinical requirements for blood components cannot be met in a timely and affordable manner in at least 75% of cases where transfusion is needed [2]. Blood deserts exist mostly in rural communities of low‐ and middle‐income countries (LMICs), where the nearest blood bank may be hours away. Each year, hundreds of millions of people go through traumatic injuries, perioperative bleeding, obstetric haemorrhage and anaemias, which require immediate medical and surgical assistance with blood transfusion. Haemorrhage is responsible for 40% of deaths among trauma patients and approximately 30% of maternal deaths globally among those who do not receive adequate transfusion [3, 4]. While clinicians in most high‐income countries have a relatively straightforward approach towards transfusion management, finding a timely supply of blood products for transfusion might be challenging in LMICs [5, 6]. Undoubtedly, the safe transfusion of blood components is only one aspect of the entire blood transfusion continuum, which includes interrelated steps ranging from donor selection to adverse event monitoring and population health. Each of these steps depends on infrastructure, trained human resources, adequate logistics and financial compatibility.

Developing access to safe blood is fundamental to promoting a horizontal healthcare system in any community [7]. The unavailability of blood continues to be one of the most significant challenges in global health and the establishment of a robust healthcare model. Achieving the 2030 goal for universal health is not possible without a sustainable transfusion ecosystem [8]. However, the final step of developing any such system involves efficient supply chain management for timely delivery of blood and blood products. But it remains a critical issue in LMICs because of multiple economic, environmental and social factors. The challenges include, but are not limited to, mismatches between supply and demand, decentralized transfusion services and inadequate infrastructure. Therefore, in addition to traditional blood delivery systems, innovative solutions such as unmanned aerial vehicles (UAVs) or drone‐based delivery could be explored to improve the capacity and efficiency of blood supply in LMICs.

The European Union Aviation Safety Agency (EASA) [9] and the Federal Aviation Agency (FAA) [10] define a ‘drone’ or ‘UAV’ as ‘any aircraft operating or designed to operate autonomously or to be piloted remotely without a pilot on board’. UAVs use software‐controlled flight plans and navigation in their embedded systems; on‐board components such as antennas, receivers, cameras, speed and flight controllers, altimeters and ultrasonic and collision sensors that prevent them from crashing during flights [11]; and a global positioning system (GPS). They can provide rapid delivery of blood products in the absence of extensive blood banking [12]. Moreover, they can leverage advanced GPS technology to navigate various terrains accurately.

In this brief review, we first outline the various types of UAVs available for use, along with their respective configurations and regulatory requirements. Then we summarize the current challenges of blood availability in resource‐limited regions. Finally, we try to explore potential pathways for significantly enhancing blood accessibility for patients in LMICs through the strategic implementation of UAVs. In doing so, we consider the various barriers and facilitators related to this innovative approach.

## DIFFERENT TYPES OF UAVs AND DELIVERY MODELS

**FIGURE 1:**
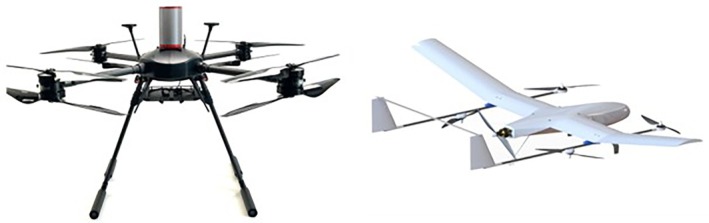
Structure of a multi‐copter unmanned aerial vehicle (UAV) (left) and vertical take‐off and landing (VTOL) aircraft systems (right).

The current UAVs are equipped with autonomous flight systems that optimize the efficiency of each delivery, ensuring that blood supplies reach their intended destinations swiftly and reliably. In brief, UAVs fall into two main categories: either with rotor or with fixed‐wing, depending on their purpose. The former includes tricopters, quadcopters, hexacopters and octocopters. The latter consists of hybrid vertical take‐off and landing (VTOL) aircraft systems. In either case, they do not need a wheeled take‐off, which is necessary, for instance, for airplanes or helicopters. Figure 1 shows two typical UAVs, and Table 1 compares their features. The example describes drones used by a company operating in Africa, which have the capacity to carry 1.75 kg (3.85 lb) and can fly at speeds of 101 km/h (62.75 mph) at altitudes ranging from 80 to 120 m above ground level [13]. Each drone can carry a maximum of three blood products in one flight. The drone at the launch site is set in motion by a supercapacitor‐powered electrical catapult launcher, and at the end of the flight, a hook is used to capture and decelerate the drone. The drone, when near the delivery site, lowers its altitude to about 25–30 m and offloads the blood product by airdropping (using a parachute) within a 5‐m drop radius. Alternately, drone‐based blood delivery systems can adopt a ‘land‐and‐deliver’ model. Drop‐only systems are faster and reduce ground‐time risks but may limit package security and traceability, while land‐based delivery enables precise handover, chain‐of‐custody assurance and return flights [14]. With real‐time tracking and monitoring integration, operators can maintain a seamless connection between the distribution centre and the receiving location. Moreover, it allows for timely updates and enhanced accountability throughout the delivery process. Table 2 summarizes information on UAV manufacturers, configurations, delivery methods and applications in LMICs.

**TABLE 1:** Comparison of multi‐copter and vertical take‐off and landing unmanned aerial vehicles, highlighting their suitability for blood delivery in low‐ and middle‐income countries.

Feature	Multi‐copter UAVs	VTOL UAVs
Take‐off and landing	Ideal for constrained or improvised sites	VTOL combined with efficient fixed‐wing cruise phase
Energy efficiency	Lower aerodynamic efficiency; reduced endurance for long‐range missions	Higher cruise efficiency, significantly extended range and reduced energy consumption
Payload stability	Stable hover and precise positioning; suitable for delicate medical payloads	Stable in cruise but less accurate in hover; may require advanced control for sensitive payloads
Operational complexity	Simple to operate and maintain; minimal pilot training required	Higher system and operational complexity; increased pilot and maintenance requirements
Cost considerations	Lower acquisition and maintenance cost; widely available platforms	Higher capital and operational cost due to hybrid architecture
Suitability for LMICs	Highly suitable where infrastructure is limited and delivery distances are moderate	Suitable for long‐range interfacility missions, less ideal where technical support is limited

Abbreviations: LMICs, low‐ and middle‐income countries; UAV, unmanned aerial vehicle; VTOL, vertical take‐off and landing (aircraft systems).

**TABLE 2:** Summary on unmanned aerial vehicle manufacturers, configurations, delivery methods and applications in low‐ and middle‐income countries.

UAV/model name	Manufacturer/country of origin	Type of configuration	Range (km)	Pay‐load (kg)	Cruising speed (km/h)	Delivery mode	LMIC countries of documented use	Medical applications	Engineering/operational notes
Zipline Sparrow (Zip 2)	Zipline Inc.—USA/Rwanda/Ghana	Fixed‐wing (catapult launch, parachute delivery)	~160 km round‐trip	1.8	100–120	Catapult launch, parachute drop	Rwanda, Ghana, Nigeria, Kenya	Blood, vaccines, emergency drugs	Highly reliable long‐range UAV; requires dedicated launch and recovery sites; not VTOL
Wingcopter 178	Wingcopter GmbH—Germany	Convertible VTOL + fixed‐wing	120	6	150	Variable	Malawi, Vanuatu	Blood, pharmaceuticals, diagnostics	Hybrid VTOL design; efficient and precise; higher acquisition and maintenance costs
Swoop Aero Kookaburra	Swoop Aero—Australia	VTOL fixed‐wing	175	3	100	Variable	Malawi, Mozambique, DR Congo	Blood, vaccines	Fully autonomous network‐based operations; requires GSM/satellite connectivity
Matternet M2	Matternet—Switzerland	Multirotor (quadcoptor)	20	2	70	Variable	Malawi, Philippines	Urban delivery of blood, samples	Compact and accurate; suited for short‐range urban missions; limited endurance
RigiTech Eiger	RigiTech—Switzerland	Fixed‐wing VTOL	100	3	90	Variable	Kenya, Rwanda (pilot)	Blood, diagnostic samples	Modular composite airframe; fast battery swap; under regulatory assessment
ABzero Smart Capsule	ABzero Srl—Italy	Modular multirotor (integrated smart payload capsule)	20–40	4–5	60–80	Variable	Pilot trials in Italy; partnerships in Africa and Middle East under development	Blood, plasma, organs, biologicals	Intelligent ‘smart capsule’ monitors temperature, vibrations, and chain‐of‐custody; can integrate with existing emergency systems; short–medium range; strong human–UAV interface design
OX DelivAir (Prototype)	OX DelivAir—UK	Multirotor	10–15	1	50	Variable	Sub‐Saharan Africa (pilot)	Emergency deliveries (AED, blood)	Urban point‐to‐point concept; low payload and range
Delivery Drones Africa (local models)	Local manufacturers—Nigeria, Uganda, Tanzania	Fixed‐wing/ multirotor	30–80	1–5	60–100	Variable	Nigeria, Uganda, Tanzania	Blood, basic medicines	Locally produced low‐cost UAVs; variable reliability and safety standards
Vertiplane ×3	TechEagle‐India	Convertible VTOL	100	3	120	Land on‐site	India (north‐east)	Blood, pharmaceuticals, diagnostics	Locally produced low‐cost UAVs; variable reliability and safety standards
i‐drone	Indian Council of Medical Research, India	Fixed‐wing VTOL	40	3–5	70–75	Land on‐site	India	Blood, vaccines, emergency drugs	Locally produced low‐cost UAVs; variable reliability and safety standards

Abbreviations: AED, automated external defibrillator; LMICs, low‐ and middle‐income countries; UAV, unmanned aerial vehicle; VTOL, vertical take‐off and landing (aircraft systems).

## REGULATORY GUIDELINES FOR UAVs

Extensive efforts have been made to facilitate the integration of UAVs into the medical field, particularly through the creation of a global regulatory framework. Europe, for example, has established a legal framework called U‐space, whose regulations have been in force since 26 January 2023. U‐space is designed to safely and efficiently integrate UAVs into the airspace, utilizing an automated management system that enables beyond‐visual‐line‐of‐sight (BVLOS) operations, which are essential for applications such as medical deliveries, emergency response and urban air mobility. Using advanced technologies like automated flight approval, real‐time traffic control and conflict resolution, U‐space keeps the airspace safe and reduces crowding, making it easier to use UAVs widely while following national aviation rules. The United States, while not having a direct equivalent to U‐space, is developing its own approach to UAV integration. The FAA has implemented regulations like Part 107, which governs small drone operations, and is working towards integrating UAVs into the National Airspace System (NAS) through initiatives like the Unmanned Aircraft System (UAS) Integration Pilot Programme and the subsequent BVLOS rule. In LMICs such as India, the i‐DRONE (Indian Council of Medical Research Drone Response and Outreach for North East) project has been launched to evaluate UAV usage in difficult terrains and in disaster management. These programmes aim to address the challenges of safely managing increasing aerial traffic, particularly for complex UAV operations like those envisioned for medical deliveries, and are exploring similar technological solutions to those employed by U‐space, such as remote identification and traffic management systems. Specific regulatory approaches and technological solutions may vary from country to country; however, the common goal is to harness the potential of UAVs to enhance healthcare delivery, particularly in areas where traditional transportation methods are limited or inefficient. The increasing global effort to integrate UAVs safely and effectively into the airspace is paving the way for a future where medical drones would play a crucial role in improving healthcare access and outcomes worldwide.

## CURRENT CHALLENGES OF BLOOD AVAILABILITY IN LMICs

While blood is an essential therapeutic, the transfusion ecosystem required to deliver safe and timely blood remains complex and challenging in LMICs. The challenges are primarily concerned with blood availability, blood testing and storage as well as the transportation of blood and blood products. Following successful blood collection and appropriate testing, proper and timely administration of blood products to recipients relies on adequate human resources powered by necessary infrastructure and integrated logistics.

### Lack of a national blood policy

The major challenge in achieving timely access to blood and blood products in LMICs is the absence of a clear national policy for strategic and systematic blood product delivery. According to a recent World Health Organization (WHO) report, 66%—or 113 out of 171—of reporting countries have specific legislation covering the safety and quality of blood transfusion, including 63% and 39% from middle‐income and low‐income countries, respectively [15]. Blood products and related therapeutic substances derived from the human body should not just be considered commodities. Beyond technical assessment, pragmatic considerations are crucial before drafting government health policies [16]. Assessing the true blood requirements in a community would require collaboration among partners from multiple medical and surgical specialties; epidemiological, statistical and supply chain sciences; and economics and social sciences [17]. It is imperative to note that most of the LMICs fail to identify blood products as national resources. Alternative blood donation strategies, driven by economizing efforts, are increasingly jeopardizing the safety of blood transfusions by undermining community solidarity and social cohesion. There is a persistent concern that a parallel paid donation system could disrupt the sustainable supply of safe blood products by challenging existing systems that are founded on the customary voluntary, non‐remunerated blood donation practices. To overcome these limitations, LMICs should start identifying the local priorities using implementation science tools and by following a model such as the BLOODSAFE programme in sub‐Saharan Africa [18].

### Supply–demand mismatch

Changing population demographics have altered the landscape of blood transfusion. Intriguingly, more than 70 countries globally, primarily among the LMICs, have a blood donation rate much less than the necessary baseline requirements [19, 20]. Considering the growing need for blood and blood products every year, it is the right time for these countries to embrace a national capacity‐strengthening strategy to meet the need for blood components and related plasma‐derived medicinal products. For example, haemoglobinopathies and malaria remain the major reasons for regional blood use in sub‐Saharan Africa [18]. However, the unmet need for surgery has increased to 160 million procedures per year, and complex surgeries such as oncosurgeries are now being performed more frequently in LMICs than the 2015 data on global surgery [21]. Moreover, the COVID‐19 pandemic led to prolonged disruptions in blood supply chain activities, exacerbating the already fragile blood systems in LMICs and resulting in extensive delays to patient care [22]. In South Asian countries, including India, family or replacement donations still provide blood for patients [23]. Such systems are unreliable, putting the onus of providing blood on the patient's families rather than on the health system. To overcome this barrier, national regulatory authorities should develop appropriate strategies to improve the overall blood supply system in LMICs.

### Inadequate blood testing facilities for transfusion‐transmissible infections

At least 47% of donations in low‐income countries and 18% in middle‐income countries fail to access advanced screening strategies, reflecting a wide gap in residual risk for transfusion‐transmissible infections (TTIs) between developed and developing countries [24]. To minimize these risks, governments imposed stringent regulations to ensure blood safety and prevent TTIs. However, the requirements for setting up blood banks in remote areas are often challenging to meet because of inadequate infrastructure and a shortage of trained personnel. These issues highlight the importance of strategic planning, risk assessment and a pragmatic approach to regulatory compliance considering the limitations of LMICs.

### Inappropriate use and wastage of blood products

National data on the use of blood products are limited, but a few studies suggest that blood products are often misused in LMICs [25, 26]. Not adhering to the transfusion guidelines remains a major concern in LMICs. Therefore, a comprehensive policy on patient blood management should be publicly available for transfusion practices [27]. Additionally, there is evidence that in LMICs, a significant amount of blood and plasma‐derived products go unused and are ultimately discarded. This primarily occurs because of inadequate infrastructure for long‐term temperature‐controlled storage and an ineffective transfer system to nearby facilities [28]. Deviations from the recommended temperature ranges can lead to irreversible damage to the stored blood, leading to life‐threatening transfusion reactions [29]. Temperature control is crucial for the transportation of blood and blood components to avoid the potential risk of bacterial contamination and haemolysis, especially when they are exposed to hostile conditions or warm environments. Therefore, maintaining cold chain integrity is essential for the entire blood supply chain, from collection to transfusion [30]. Furthermore, remote areas in LMICs are often reliant on limited road infrastructure, leading to difficulties in accessibility and transfer of blood and blood products. Considering these major limitations in the blood delivery continuum, it is imperative that new technologies should be implemented, for instance, UAVs for emergency transfusion in rural regions.

## ROLE OF UAVs TO IMPROVE THE BLOOD DELIVERY SYSTEM IN LMICs

Integrating UAVs for blood delivery within the medical sector is significantly transforming the speed and efficiency of logistics, particularly for distributing blood and blood products to remote or challenging disaster‐affected areas in LMICs. The delivery of these critical products encountered considerable obstacles due to damaged or inadequate infrastructure, impassable roads and unpredictable weather conditions, resulting in potentially life‐threatening delays. A study conducted in Rwanda found that UAVs were, on average, 79 min faster than traditional driving times for transporting blood and blood products. However, the time savings from using UAVs varied widely, ranging from 3 to 211 min across different facilities [31].

The WHO categorizes hospital blood needs into three levels: emergency, certain need and possible need [24]. Emergencies are particularly critical due to tight time constraints (requiring blood within 60 min) and the high demand for the universal blood group O. Urgent transport is costly and risks blood quality degradation. By optimizing aerial delivery routes, UAVs can ensure multiple and precise deliveries within a compressed timeframe. Furthermore, UAVs can be specifically designed to ensure the delivery of blood supplies under various weather conditions, owing to their robust and weather‐resistant characteristics. This capability enhances the efficiency of medical supply chains, enabling timely access to critical resources regardless of environmental challenges. Such technological advancements provide a significant advantage in emergencies, where the prompt availability of blood products can be vital for patient care and outcomes. For example, postpartum haemorrhage remains the leading cause of death for pregnant women in LMICs, and a significant number of blood units are required to prevent complications in childbirth [4]. In 2023, UAVs delivered a total of 28,754 units of blood to patients in Rwanda, the majority of whom were women in critical moments of delivery [13]. Incorporating UAVs into blood delivery systems has significantly enhanced the ability to maintain optimal conditions for blood products. The precise temperature control and adherence to specific storage protocols can be effectively managed through the sophisticated design of these UAVs [32]. Despite stringent regulations, studies indicate that over 40% of blood transports in LMICs fail to adhere to established temperature guidelines [33, 34]. This jeopardizes blood quality, resulting in increased costs (due to wastage and additional testing) and, most importantly, posing serious risks to patient safety.

**FIGURE 2:**
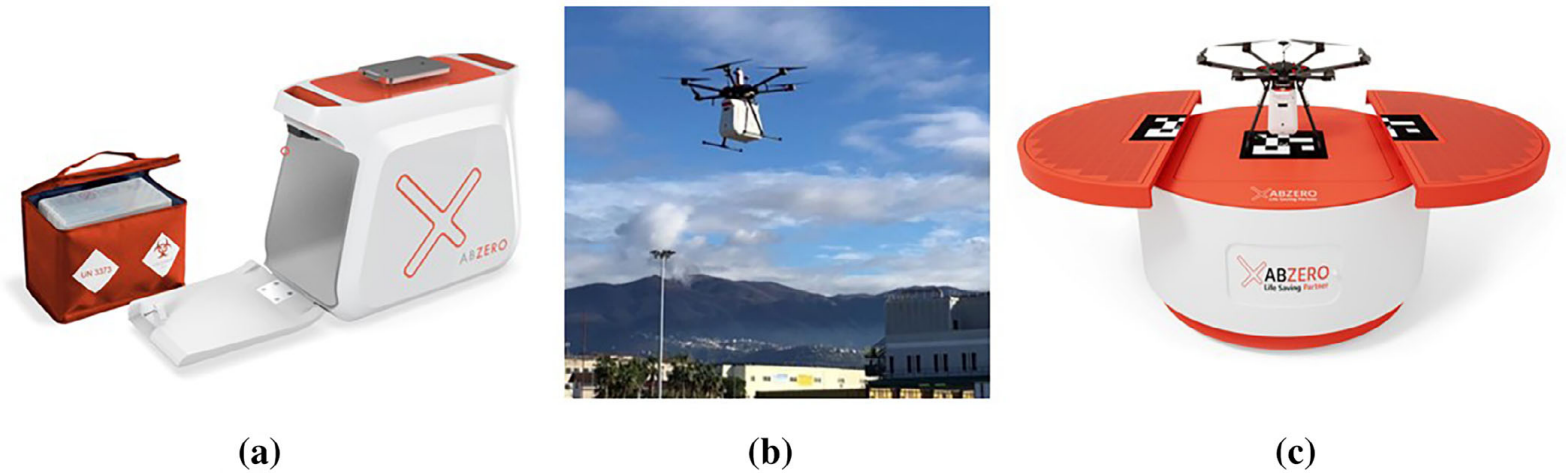
(a) Certified container and Smart Capsule for blood transportation. (b) A coaxial quadcopter carrying the Smart Capsule. (c) Drone landing pad.

Developing a new smart capsule, equipped with artificial intelligence (AI), can ensure the optimal temperature control of blood and blood products during drone transport. One such smart capsule has been designed recently in compliance with the 2002/98/EC directive, which sets quality and safety standards for the collection, testing, processing, storage and distribution of blood components in the European Union (EU) [35]. Next‐generation modular stabilizers were used for the transport and storage of heat‐labile blood products at controlled temperatures. The temperature stabilizers are available in three versions: for transport of red cells, platelets and plasma at +4, +22 and −30°C, respectively. Temperatures up to −80°C can be maintained using dry ice. Real‐time temperature monitoring is done by the Plasma Check System, which employs a time‐strip sensor affixed to the bag and a data logger. An alert system is in place to detect an early temperature increase. Medical staff can attach the capsule to a UAV and track its flight with a user‐friendly mobile app [36, 37]. Figure 2 shows images of the smart capsule for blood delivery.

Using UAVs lowers the risk of environmental contamination and reduces product degradation, thereby providing substantial benefits for patients needing blood transfusions. Consequently, UAV‐based delivery represents an innovative solution to strengthen the blood supply chain, particularly in critical emergencies such as trauma care, maternal health and transfusions for individuals with rare blood types [38]. Furthermore, massive transfusion could be successfully performed in a combat environment using UAV‐transported blood products [39]. The timely delivery of blood products using UAVs, combined with remote medical guidance, has the potential to change the landscape of pre‐hospital care and disaster response despite extreme conditions and delayed evacuation. Another significant advantage is that blood plasma and platelets might be made available to hospitals in remote locations without temperature‐controlled freezers, thereby reducing the number of patients who are needlessly referred to higher facilities solely for transfusions.

It is essential to acknowledge that employing UAVs in blood delivery systems presents a safe and environmentally friendly alternative to traditional methods. Conventional blood delivery often relies on vehicles, contributing to air pollution and greenhouse gas emissions, posing a significant environmental threat [40]. In contrast, integrating electric or hybrid UAVs in the medical sector results in almost zero emissions, making it a sustainable choice. Moreover, these advanced UAVs reduce harmful pollutant release and significantly reduce noise pollution, creating a quieter urban landscape. The use of UAVs also helps in reducing the waste generated during blood delivery, resulting in a more efficient and environmentally friendly approach within the healthcare sector. This innovative method promises to streamline logistics while prioritizing the health of our planet.

Last but not least, while conducting a generalized cost analysis is complicated due to varying regional expenses for staff and transportation, the significant reduction in transit times and the deployment of specialized healthcare professionals are expected to result in substantial cost savings for national health systems. It allows the reallocation of healthcare personnel from ambulance duty to more specialized and impactful roles within healthcare facilities in LMICs. Establishing even a small blood bank necessitates significant investment, encompassing equipment (centrifuges, freezers, labelling systems, laboratories and diagnostic tools), consumables (collection bags, test tubes and reagents), IT infrastructure (management software, hardware and installation) and operational costs (utilities, maintenance, waste disposal and transportation). Staffing (doctors, nurses and laboratory technologists) and regulatory compliance (authorizations and accreditations) also contribute to the cost. These combined investments could vary significantly, depending on the purchasing power parity of different countries [41, 42]. Furthermore, these costs do not include expenses associated with facility upkeep and the provision of related services. On the contrary, the purchase of a UAV and the related licences can be considered a one‐time investment. It is worth noting that maintaining central blood banks in urban areas is important for creating an efficient blood distribution network, as there are typically more donors available compared to rural regions.

## REPORTING FROM A BLOOD DESERT ZONE IN INDIA

**FIGURE 3:**
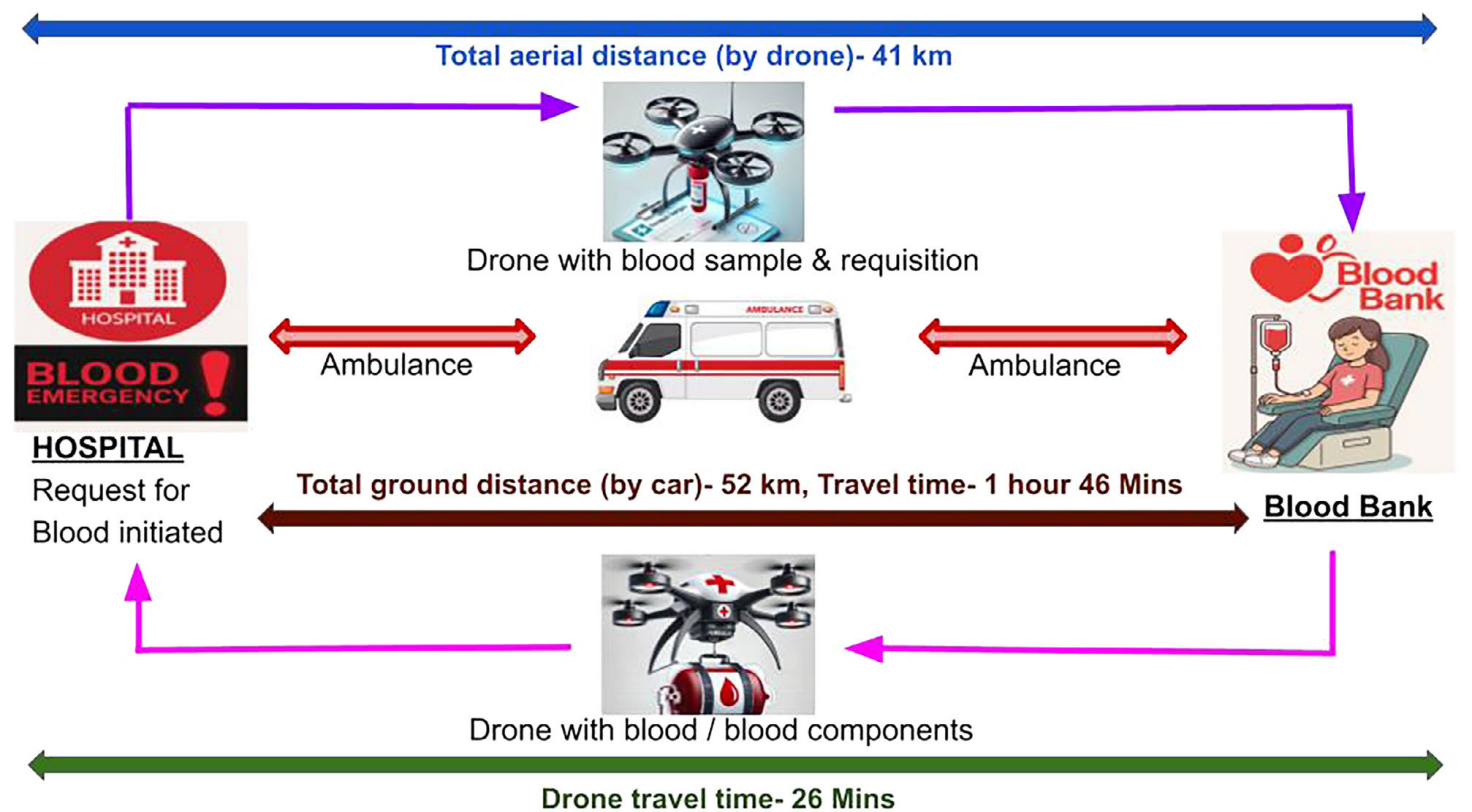
Schematic diagram of the unmanned aerial vehicle (UAV) pathway for the delivery of blood and blood components with distances (ground and aerial) and transportation time.

In a recent study conducted by Kundu et al. [23] and subsequently expanded by Dutta et al. [4] examining the conditions faced by residents of Bihar, a state in India with approximately 130 million citizens located in one of the country's most economically disadvantaged regions, it was demonstrated that the caesarean section rate between 2018 and 2019 performed at the First Referral Units (FRUs) was 3.1%, significantly below the rates recommended at the state, national and international levels; the WHO minimum recommended rate is 10% [43]. The low rate was attributed to the fact that these FRU facilities are located in a ‘blood desert’ zone. Many facilities that provide essential maternal health services, such as vaginal delivery, struggle to obtain immediate access to blood. Terrestrial transportation presents significant logistical challenges, especially in regions with inadequate infrastructure. The use of Walking Blood Banks (WBBs) and Intraoperative Autotransfusion (IAT) has addressed this difficulty. Unfortunately, neither has proven to be a definitive solution. The use of WBBs is discouraged because of concerns regarding the lack of regulatory provisions governing the testing process, especially for TTIs. IAT requires adequate training of healthcare professionals in the delivery room and expensive equipment and, in any case, only serves to stabilize the patient until definitive treatment is available. In regions without blood banks with adequate storage facilities and human logistics, there could be adoption of blood transport systems based on UAVs carrying blood. Figure 3 illustrates how the rapid delivery of essential blood products using UAVs could significantly improve the hospital's response in an emergency situation.

## BARRIERS AND LIMITATIONS OF IMPLEMENTING UAVs FOR BLOOD DELIVERY

UAV‐based blood delivery implementation comes with its own set of challenges despite its potential. Because of the high cost of these services and operations, they require proper plans to reduce overall costs without compromising the benefits [44]. Although venture capital investments are encouraged for UAVs, creating self‐sufficient combined public–private funding structures is essential for maintaining these infrastructures in the long run. The financial models must be sustainable because of heavy government influence over the implementation of this technology. The hostile political environment significantly affects sustainability in many LMICs. One government may support the implementation of UAVs to enhance blood delivery, while the next may be unwilling to invest the necessary resources. A viable mathematical model for assessing the costs associated with drone‐based blood delivery should be developed before implementation in LMICs, as it might enhance funding opportunities.

Delivery of blood using UAVs could be risky in areas with insurgency and armed conflicts. For example, commercial transportation using UAVs is not allowed closer than 25 km from the international border in India for security reasons [45]. However, the creation of such guidelines is influenced by several geopolitical factors within a region. Moreover, community support and awareness are necessary for successful implementation of UAV‐based blood delivery in remote areas.

Blood delivery through UAVs might provide a false impression of the availability of blood donors and inventory. Utilizing UAVs to deliver blood to remote areas eventually increases access to blood supply, leading to increased blood demand. Exacerbation of this unmet need is crucial in the context of the existing blood shortages, which may further stress the blood banks with limited blood stocks.

## CONCLUSIONS

The use of UAVs in medicine is becoming increasingly crucial, especially in LMICs for blood delivery, where blood banks are concentrated in major urban areas, leaving smaller communities and rural areas with limited or no access. In nearly all cases, blood bags for transfusions are transported from blood banks to the peripheral healthcare facilities via ambulances, which are significantly constrained by the condition of road infrastructure and traffic, often rendering timely medical intervention impossible. To enhance cost effectiveness, it would be beneficial to utilize longer range UAVs that offer superior geographical coverage. Furthermore, building commercial partnerships for carrying various products on the return flight could reduce the operational cost. The issue of temperature regulation is critical when considering the changing global climate. Further research is needed to evaluate the maintenance of the quality of blood components in equatorial weather. Nesting the mid‐sized hospitals could significantly improve the blood distribution network. Moreover, efficacy studies need to be expanded past delivery time to evaluate effects on patient outcomes. These clinical outcomes include, but are not limited to, overall survival, disease‐specific morbidity, maternal and infant mortality rates and disability‐adjusted life years saved in a given time frame with one functional UAV. These should be compared with the local standards of care to understand the actual impact of UAV‐based blood delivery in resource‐limited settings. It has the potential to improve overall healthcare facilities and health‐seeking behaviour of the population. Finally, UAV‐based delivery should go along with a mature blood transfusion system, and adequate investment should be made in strengthening existing voluntary blood donation programmes in LMICs to improve the availability of blood and blood products.

## CONFLICT OF INTEREST STATEMENT

Giuseppe Tortora is the founder and CEO of ABzero, designed for aerial drone transport of biomedical products. All other authors declare no conflicts of interest.

## Data Availability

The data that support the findings of this study are available on request from the corresponding author. The data are not publicly available due to privacy or ethical restrictions.
